# Relationships Between Age, Frailty, Length of Care Home Residence and Biomarkers of Immunity and Inflammation in Older Care Home Residents in the United Kingdom

**DOI:** 10.3389/fragi.2021.599084

**Published:** 2021-03-17

**Authors:** Vivian M. Castro-Herrera, Mark Lown, Helena L. Fisk, Eleri Owen-Jones, Mandy Lau, Rachel Lowe, Kerenza Hood, David Gillespie, F. D. Richard Hobbs, Paul Little, Christopher C. Butler, Elizabeth A. Miles, Philip C. Calder

**Affiliations:** ^1^ School of Human Development and Health, Faculty of Medicine, University of Southampton, Southampton, United Kingdom; ^2^ School of Primary Care and Population Sciences, Faculty of Medicine, University of Southampton, Southampton, United Kingdom; ^3^ Centre for Trials Research, Cardiff University, Cardiff, United Kingdom; ^4^ Nuffield Department of Primary Care Health Sciences, University of Oxford, Oxford, United Kingdom; ^5^ NIHR Southampton Biomedical Research Centre, University Hospital Southampton NHS Foundation Trust and University of Southampton, Southampton, United Kingdom

**Keywords:** care home residents, aging, frailty, immunity, inflammation, immunosenescence, inflammageing

## Abstract

Aging is associated with changes to the immune system, collectively termed immunosenescence and inflammageing. However, the relationships among age, frailty, and immune parameters in older people resident in care homes are not well described. We assessed immune and inflammatory parameters in 184 United Kingdom care home residents aged over 65 years and how they relate to age, frailty index, and length of care home residence. Linear regression was used to identify the independent contribution of age, frailty, and length of care home residence to the various immune parameters as dependent variables. Participants had a mean age (±SD) of 85.3 ± 7.5 years, had been residing in the care home for a mean (±SD) of 1.9 ± 2.2 years at the time of study commencement, and 40.7% were severely frail. Length of care home residence and frailty index were correlated but age and frailty index and age and length of care home residence were not significantly correlated. All components of the full blood count, apart from total lymphocytes, were within the reference range; 31% of participants had blood lymphocyte numbers below the lower value of the reference range. Among the components of the full blood count, platelet numbers were positively associated with frailty index. Amongst plasma inflammatory markers, C-reactive protein (CRP), interleukin-1 receptor antagonist (IL-1ra), soluble E-selectin and interferon gamma-induced protein 10 (IP-10) were positively associated with frailty. Plasma soluble vascular cell adhesion molecule 1 (sVCAM-1), IP-10 and tumor necrosis factor receptor II (TNFRII) were positively associated with age. Plasma monocyte chemoattractant protein 1 was positively associated with length of care home residence. Frailty was an independent predictor of platelet numbers, plasma CRP, IL-1ra, IP-10, and sE-selectin. Age was an independent predictor of activated monocytes and plasma IP-10, TNFRII and sVCAM-1. Length of care home residence was an independent predictor of plasma MCP-1. This study concludes that there are independent links between increased frailty and inflammation and between increased age and inflammation amongst older people resident in care homes in the United Kingdom. Since, inflammation is known to contribute to morbidity and mortality in older people, the causes and consequences of inflammation in this population should be further explored.

## Introduction

The number and proportion of older people is increasing in many societies (GBD 2016 Causes of Death Collaborators, 2017; GBD 2016 DALYs and HALE Collaborators, 2017). Aging increases the risk of morbidity, bringing with it loss of independence, increased health and social care costs, and for many older people, the need to move to a care home. Aging is also associated with changes to the immune system, collectively termed immunosenescence (De Martinis et al., 2004; Agarwal and Busse, 2010; Pawelec et al., 2010; Castelo-Branco and Soveral, 2014; Bektas et al., 2017) and inflammageing (Franceschi, 2007; Calder et al., 2017; Ventura et al., 2017; Atienza et al., 2018). Immunosenescence involves changes in the numbers of different immune cells in the bloodstream and reductions in their function (De Martinis et al., 2004; Agarwal and Busse, 2010; Pawelec et al., 2010; Castelo-Branco and Soveral, 2014; Bektas et al., 2017). For example, there is reduced production and export of naïve T lymphocytes into the blood (and lymphoid tissues) during aging with a loss in T cell receptor diversity and an accumulation of memory T lymphocytes (Agarwal and Busse, 2010; Pawelec et al., 2010; Castelo-Branco and Soveral, 2014; Bektas et al., 2017). The overall result of these changes are lowered numbers of T lymphocytes in the blood and impaired T lymphocyte responsiveness (De Martinis et al., 2004; Agarwal and Busse, 2010; Pawelec et al., 2010; Castelo-Branco and Soveral, 2014; Bektas et al., 2017). Immunosenescence also affects B lymphocyte numbers and function and the function of antigen presenting cells and some components of innate immunity (De Martinis et al., 2004; Agarwal and Busse, 2010; Pawelec et al., 2010; Castelo-Branco and Soveral, 2014; Bektas et al., 2017). Inflammageing is seen as an increase in blood plasma or serum concentrations of the acute phase protein C-reactive protein (CRP) and of inflammatory cytokines like interleukin (IL)-6 (Franceschi, 2007; Calder et al., 2017; Ventura et al., 2017; Atienza et al., 2018). This may reflect sensitized pro-inflammatory signaling pathways in older people. Together these changes contribute to the increased prevalence and severity of infections (Yoshikawa, 2000; Pera et al., 2015), the poorer responses to vaccinations (Goodwin et al., 2006; Trzonkowski et al., 2009; Derhovanessian and Pawelec, 2012) and the increased likelihood to suffer illness and disability (Onder et al., 2012) that occur with aging. However, aging is heterogeneous and occurs at different rates in different individuals; different settings may influence the aging process, for example by providing different access to a good diet, physical activity, mental stimulation and social interactions. It is described that free-living older individuals have a significantly better quality of life when compared with older people in institutional care homes (Montoya and Mody, 2011; Olsen et al., 2016; Sampson et al., 2019). This may relate to the different experiences offered outside and inside care homes which may themselves contribute to the aging process.

Frailty is currently recognized as a ”geriatric syndrome” (Laksmi, 2014; Chen et al., 2016). Categorization of frailty has traditionally been according to physical mobility and strength (Dent et al., 2016), although there is also a cognitive component to frailty as recognized in some scales for evaluating the extent of frailty among older people (Morley et al., 2013). Frail older adults are at increased risk of adverse health outcomes, including falls, hospitalization, and mortality (Beck-Sague et al., 1994; Ahmed et al., 2007). It has been suggested that one of the important pathways of frailty development is the immune/inflammatory pathway (Wilson et al., 2017). Inflammation has also been linked to a wide range of chronic diseases of common prevalence within older populations (Franceschi, 2007; Bauer and Fuente, 2016). Age, frailty, and length of care home residence might be linked to adverse outcomes (Carneiro et al., 2017). In order to better understand the relationships of age, frailty and length of care home residence with immunosenescence and inflammageing, we measured a range of immune and inflammatory markers in 184 United Kingdom care home residents aged over 65 years and investigated the relevant associations. We assessed static measures in blood (full blood count, immune phenotypes, plasma immune mediator concentrations, plasma CRP) as well as blood immune cell responses after *ex vivo* challenge (phagocytosis, blood culture responses to immune stimulation) and included components of both innate and acquired immunity. Many of these markers have not been well explored in the context of aging or frailty or in older people in the care home setting.

## Methods

### Participants

This cross-sectional study is embedded within the “Probiotics to reduce infections in care home residents” (PRINCESS) trial which is a two-arm double-blind individually randomized controlled trial, involving three academic centers in the United Kingdom (Universities of Cardiff, Oxford and Southampton). The full protocol (Owen-Jones et al., 2019) and the main outcomes (Butler et al., 2020) of the PRINCESS trial have been published. The PRINCESS trial was approved by the Wales REC 3 (15/WA/0306) and is registered at www.controlled-trials.com as ISRCTN16392920. Care home residents were eligible for participation if they were aged 65 years or older and willing and able to give informed consent for participation; if they lacked capacity, a consultee could complete a consultee declaration for participation on their behalf. Exclusions were immunocompromize (ongoing immune-suppressants; long-term, high-dose, oral, intramuscular or intravenous steroids), lactose intolerance, taking ongoing regular probiotics, or temporary residence in the care home. Care homes were residential, nursing or mixed. Here we report frailty and immune parameters in a subset of participants whose data was available at study entry (*n* = 184, although not all immune parameters were available for all these participants). Data were not available for all participants in the main PRINCESS trial and in this sub-study because 1) participants did not consent to take part in the immune sub-study of PRINCESS; or 2) insufficient blood was collected to measure some or any of the immune parameters; or 3) the blood arrived at the University of Southampton, where immune measurements were made, outside of a time window pre-determined based upon an earlier study (Castro-Herrera et al., 2018).

### Assessment of Frailty

Frailty index was determined according to the scale described elsewhere (Morley et al., 2013). The scale has nine categories defined as: 1 = Very fit for their age (active, energetic and motivated); 2 = Well (absent symptomatology of disease but less active); 3 = Managing well (medical problems under control but not regularly active); 4 = Vulnerable (symptoms that limit activities but not decedent on others); 5 = Mildly frail (impairment of daily activities); 6 = Moderately frail (progressive impairment and declined activities); 7 = Severely frail (completely dependent cognitively or physically but not terminally ill); 8 = Very severely frail (completely dependent and approaching the end of life); 9 = Terminally ill (life expectancy < 6 months).

### Measurement of Immune Parameters

Blood was collected into EDTA or heparin at the care homes and was posted to the University of Southampton. Whole blood was used to determine full blood count, for immune phenotyping, for assessment of neutrophil and monocyte phagocytosis, and for cultures to determine production of immune mediators after stimulation. Plasma was prepared for measurement of CRP and immune mediator concentrations. Immune parameters were measured as described in detail previously (Castro-Herrera et al., 2018). Briefly, full blood count was determined in blood collected into EDTA using an automated UniCel Beckman Coulter Dxl 800 (Beckman Coulter, High Wycombe, United Kingdom). Full blood collected into heparin was used for immune phenotyping using flow cytometry following staining with fluorescently labelled antibodies to immune cell surface structures. Blood (500 µl) was placed in BD Trucount™ tubes (BD Pharmingen Oxford, United Kingdom). Antibodies were purchased from BD Pharmingen (Oxford, United Kingdom). Table 1 lists the details of the immune phenotyping. Staining was performed at room temperature for 20 min and protected from light. BD-FACS lysing solution (1 ml; BD Pharmingen Oxford, United Kingdom) was added and tubes were incubated for 20 min. Tubes were vortexed and placed at room temperature in a dark place. Tubes were analyzed on a BD FACS LSRF Fortessa TM X-20 Special order (BD Biosciences, San Jose, CA). Isotype controls were run at a medium flow rate. 10,000 events were collected for all samples in tubes containing Trucount beads. Beads were gated and 5,000 events were collected within the bead region. Data analyses were performed with BD FACSDiva 8.0.1 software. Instrument stability was checked daily using the cytometer setup and tracking to evaluate performance with Research Beads™ (BD Biosciences, Oxford, United Kingdom).

**TABLE 1:** Details of immune phenotyping.

Immune cell population	CD combination used to identify the population	Fluorochrome used	µl of antibody used/test
T Cells	CD45^+^CD3^+^	PE-Cy5/AF647	20/5
Helper T cells	CD45^+^CD3^+^CD4^+^	PE-Cy5/AF647/AF488	20/5/5
Cytotoxic T cells	CD45^+^CD3^+^CD8^+^	PE-Cy5/AF647/BV605	20/5/5
Activated cytotoxic T cells	CD45^+^CD3^+^CD8^+^CD25^+^	PE-Cy5/AF647/BV605/PE	20/5/5/20
Regulatory T cells	CD45^+^CD3^+^CD4^+^CD8^−^CD25^HI^CD127^LO^	PE-Cy5/AF647/AF488/BV605/*p*E/BV421	20/5/5/5/20/5
Monocytes	CD45^+^CD14^+^	PE-Cy5/PE-Cy7	20/5
Activated monocytes	CD45^+^CD14^+^CD80^+^	PE-Cy5/PE-Cy7/BV421	20/5/20
Activated monocytes	CD45^+^CD14^+^CD86^+^	PE-Cy5/PE-Cy7/PE	20/5/20
B Cells	CD45^+^CD3^−^CD19^+^	PE-Cy5/AF647/AF488	20/5/5
Activated B cells	CD45^+^CD3^−^CD19^+^CD80^+^	PE-Cy5/AF647/AF488/BV421	20/5/5/20
Activated B cells	CD45^+^CD3^−^CD19^+^CD86^+^	PE-Cy5/AF647/AF488/PE	20/5/5/20
Natural killer cells	CD45^+^CD3^−^CD16^+^	PE-Cy5/AF647/BV605	20/5/20

AF, alexa fluor; BV, brilliant violet; Cy5, cyanine 5; PE, phycoerythrin.

Phagocytic activity of blood neutrophils and monocytes toward *E. coli* was assessed in heparinsed whole blood (200 µl) using the commercially available Phagotest™ kit (Glycotope Biotechnology GmbH, Heidelberg Germany). Events (20,000) were collected using a BD FACSCalibur flow cytometer (BD Biosciences, San Jose, CA). Both the percentage of cells (neutrophils and monocytes) involved in phagocytosis and their geometric mean fluorescence intensity (reflecting the number of ingested bacteria per cell) were analyzed.

For whole blood cultures, 500 μl heparinized whole blood was diluted 1:10 in Roswell Park Memorial Institute 1640 culture medium supplemented with penicillin (50 U/ml), streptomycin (50 μg/ml) and L-glutamine (2 mM) (Sigma Aldrich, Gillingham, United Kingdom). Diluted blood (990 μl) was added to the wells of a 24-well flat-bottomed cell culture plate. Then, 10 µl of either medium, lipopolysaccharide (LPS; from *E. coli* K12 strain), peptidoglycan (PGN; from *Staphylococcus aureus*) or phytohaemagglutinin (PHA; from *Phaseolus vulgaris*) was added to the wells to obtain final concentrations of 10 μg/ml LPS, 5 μg/ml PGN or 5 μg/ml PHA respectively. Cultures were incubated for 24 h at 37°C in an atmosphere of 95% air and 5% CO_2_. Supernatants were collected by centrifuging the plate at 2000 rpm for 5 min and were then stored at −80°C for analysis. Once all supernatants were ready to be analyzed, magnetic luminex assays (Bio-Techne, R&D Systems, Abingdon, United Kingdom) were used. Analytes were measured in negative controls and in the medium after stimulation with PGN or LPS and the assay limits of detection (pg/ml) were: tumor necrosis factor (TNF-α) (0.62), interleukin (IL)-1β (0.25), IL-6 (0.38), IL-10 (2.93), and IL-12p70 (2.39). Analytes measured following stimulation with PHA were TNF-α (limit of detection (pg/ml) (1.2) and interferon (IFN-γ) (0.4). Assays were performed according to manufacturer’s instructions. Microparticles were resuspended in buffer and read using a Bio-Rad-plex Luminex Analyzer.

Plasma was prepared from 1 ml of heparinized whole blood by centrifugation at 1,500 rpm for 5 min and stored at −20°C prior to analysis. CRP, immune mediators and soluble receptors were measured by magnetic luminex assays (Bio-Techne, R&D Systems, Abingdon, United Kingdom). Analytes measured and the assay limits of detection (pg/ml) were CRP (116), TNF-α (0.54), IL-6 (0.31), IL-10 (0.24), IL-17 (1.8), IL-12p70 (2.96), IL-1ra (18), TNF receptor II (TNFRII; 0.5), monocyte chemoattract protein (MCP-1; 9.9), soluble vascular cell adhesion molecule (sVCAM-1; 238), soluble E-selectin (sE-selectin; 18.8), soluble intercellular adhesion molecule (sICAM-1; 87.9), and interferon gamma-induced protein 10 (IP-10; 1.18). Assays were performed according to manufacturer’s instructions. Microparticles were resuspended in buffer and read using a Bio-Rad-plex Luminex Analyzer.

### Statistical Analysis

As this is an exploratory study no power calculation was done. Normality of data was assessed by visual inspection of histogram distributions and by using the Shapiro Wilk and Kolmogorov-Smirnov tests. Data were not normally distributed. Thus, data are presented using median, interquartile range and percentiles. Comparisons of outcomes between sexes were made using the Mann-Whitney U test. Correlations amongst age (as a continuous variable), frailty index and length of care home residence (as a continuous variable) were investigated using Spearmans’s test. Associations between age, frailty index, length of care home residence and each immune parameter were investigated using linear regression. Multivariate analysis using linear regression models was used to examine the independent influence of age, frailty and length of care home residence on each immune parameter. All data were log10 transformed prior to conducting these analyses. Data collation and analysis were performed in SPSS version 22, Microsoft Excel and PRISM software. In all cases a value for *p* < 0.05 was taken to indicate statistical significance; no correction for multiple testing was made.

## Results

### Participants Characteristics

Table 2 shows the characteristics of the subset of participants studied here compared to those of the entire PRINCESS cohort; the characteristics are comparable. The age range of the included care home residents was 65–102 years. They had a mean age (±SD) of 85.3 (±7.5) yr and had been residing in the care home for a mean (±SD) of 1.89 (±2.16) yr at the time of study commencement (Table 2), although it is not known if they had previously resided in another care home. There were more women than men (63.4 vs 36.6%). Over one-third (40.7%) of included participants were severely frail (category 7) and 42% were moderately or mildly frail (categories 6 and 5) (Table 2). Age, frailty and duration of care home residence did not differ between women and men (data not shown).

**TABLE 2:** Characteristics of participants in this study and those of the full PRINCESS cohort at commencement of study and enrollment.

Variable	Full PRINCESS cohort				Subset participating in this study			
	*n*	Mean (SD)	Median (IQR)	Min, max	*n*	Mean (SD)	Median (IQR)	Min, max
Age (yr)	310	85.3 (7.4)	86 (81–91)	65, 102	184	83.1 (15.7)	86 (80–91)	65, 102
Length of care home residence (yr)	307	1.7 (2.4)	1 (0–2)	0, 15	184	1.8 (2.2)	1 (0.4–2.4)	0, 15
		Frequency		%		Frequency		%
Sex	310				183			
Male		103		33.2		67		36.6
Female		207		66.8		116		63.4
Frailty index	310				150			
1 (very fit)		4		1.3		1		0.7
2 (well)		8		2.6		5		3.3
3 (managing well)		19		6.1		13		8.7
4 (vulnerable)		11		3.5		7		4.7
5 (mildly frail)		20		6.5		13		8.7
6 (moderately frail)		84		27.1		50		33.3
7 (severely frail)		158		51.0		61		40.7
8 (very severely frail)		6		1.9		0		0
9 (terminally ill)		0		0		0		0

### Association Amongst Age, Frailty and Length of Care Home Residence

**FIGURE 1:**
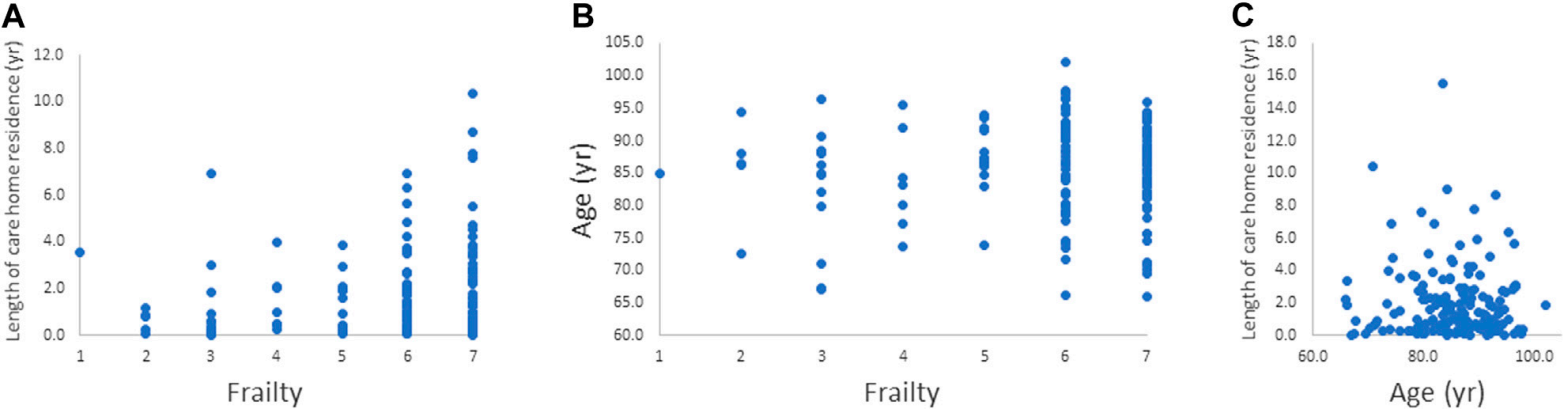
Relationships between. **(A)** frailty index and length of care home residence, **(B)** frailty index and age, and **(C)** age and length of care home residence. The relationship between frailty index and length of care home residence was significant (*p* = 0.023).

There was a significant positive correlation between length of care home residence and frailty index (Spearman’s correlation coefficient = 0.185; *p* = 0.023) as shown in Figure 1A. Age and frailty index and age and length of care home residence were not significantly correlated (Figures 1B,C).

### Full Blood Count and Immune Parameters

Data for the components of the full blood count were mainly within the reference range, apart from lymphocyte numbers (Table 3). Many participants had low blood lymphocyte numbers, with 31% (*n* = 49) having numbers below the lower value of the reference range. The percentage of women and men with lymphocyte numbers below the lower value of the reference range did not differ. Age, frailty, and length of care home residence were not different between those with blood lymphocyte numbers below or within the reference range. A small proportion of participants (*n* = 12; 7.6%) had platelet numbers above the upper value of the reference range. Platelet numbers were higher in women than men (median (10th and 90th centile) 293 (211, 389) vs 251 (168, 386) 10^9^/L; *p* = 0.039). Data for immune phenotypes, neutrophil and monocyte phagocytosis, plasma CRP and immune mediator concentrations, and concentrations of immune mediators in stimulated whole blood cultures are shown in Tables 4–7, respectively. There are no reference values for these immune outcomes, but Table 4 lists a selection of previously reported vales for immune phenotypes in older individuals (Seidler et al., 2010; Tavares et al., 2014; Qin et al., 2016). Participants in the current study had lower numbers of T lymphocytes and natural killer cells and a lower ratio of CD4^+^ to CD8^+^ T lymphocytes than reported in these other studies of older adults. Ten percent of participants had a ratio of CD4^+^ to CD8^+^ T lymphocytes less than 1 (Table 4). The only immune outcome that differed between sexes was plasma IL-10 concentration, which was higher in men than women (median (10th and 90th centile) 0.66 (0.25, 3.59) vs 0.56 (0.12, 1.64) pg/ml; *p* = 0.039).

**TABLE 3:** Full blood count results for older people resident in care homes.

Variable	Reference range (10^9^/L)	*n*	Median	10th percentile	90th percentile
			Number of cells (10^9^/L)		
Neutrophils	2.0–7.5	151	4.5	2.90	7.2
Lymphocytes	1.5–5.0	157	1.6	0.9	2.5
Monocytes	0.2–1.0	158	0.6	0.3	0.9
Eosinophils	0.0–0.5	153	0.1	0.1	0.3
Basophils	0.0–0.1	153	0.1	0	0.1
Total leukocytes	4–11	109	7.4	5.1	10.5
Platelets	140–400	158	268	191	390

**TABLE 4:** Blood immunophenotypes in older people resident in care homes along with a comparison of values from the literature.

Variable	*n*	Number of cells/μl			Tavares et al. (2014) >60 years (*n* = 35)	Qin et al. (2016) >65 years (*n* = 41)	Seidler et al. (2010) >50 years (*n* = 60)
		Median	10th percentile	90th percentile	Mean (SD) cells/μl	Mean (SD) cells/μl	Median (range) cells/μl
T Cells	148	1,249	875	1,726	1,336 (630)	1,846 (505)	—
Helper T cells	148	859	304	1,391	780 (436)	699 (281)	—
Cytotoxic cells	148	648	402	1,005	417 (313)	448 (235)	—
Activated cytotoxic T cells	142	224	126	367	—	191 (115)	—
Regulatory T cells	148	40	16	191	—	—	—
Ratio CD4^+^:CD8^+^	148	1.3	1.0	1.8	1.8 (1.3)	1.5 (1.2)	—
Monocytes	148	500	255	820	—	—	420 (165–903)
Activated monocytes (CD80^+^)	148	152	36	379	—	—	—
Activated monocytes (CD86^+^)	148	106	20	275	—	—	—
NK cells	98	81	49	116	—	448 (223)	—
B Cells	148	221	102	342	191 (122)	198 (112)	—
Activated B cells (CD80^+^)	148	119	68	213	—	—	—
Activated B cells (CD86^+^)	148	118	72	220	—	—	—

**TABLE 5:** Phagocytosis of *E. coli* by blood neutrophils and monocytes from older people resident in care homes.

Variable	*n*	Median	10th percentile	90th percentile
Neutrophils with phagocytic activity (%)	147	83.9	64.6	91.6
Geometric median fluorescence intensity (GMFI) of active neutrophils	142	256.8	158.6	378.5
Monocytes with phagocytic activity (%)	147	29.9	13.6	47.9
Geometric median fluorescence intensity (GMFI) of active monocytes	147	182.1	105.9	295.9

**TABLE 6:** Concentrations of CRP and immune mediators in plasma from older people resident in care homes.

Variable	*n*	Median	10th percentile	90th percentile
CRP (mg/L)	85	2.7	0.5	16.3
sICAM-1 (ng/ml)	95	386	208	764
IL-1ra (pg/ml)	95	1,559	705	4,644
sE-selectin (ng/ml)	95	22.8	11.3	39.8
sVCAM-1 (ng/ml)	95	791	432	1,391
MCP-1 (pg/ml)	95	356	165	691
IP-10 (pg/ml)	95	152	75	285
IL-17 A (pg/ml)	95	0.9	0.6	6.9
TNFRII (pg/ml)	95	4,072	2,119	7,963
IL-6 (pg/ml)	96	4.4	1.7	20.4
IL-10 (pg/ml)	96	0.6	0.1	1.8
TNF-α (pg/ml)	96	17.7	9.2	26.4

**TABLE 7:** Immune mediator concentrations in stimulated cultures of whole blood from older people resident in care homes.

Variable	*n*	Median	10th percentile	90th percentile
Lipopolysaccharide-stimulated cultures				
IL-10 (pg/ml)	86	2,428	473	10,780
TNF-α (pg/ml)	86	13,231	3,358	32,884
IL-6 (ng/ml)	86	47.6	15.7	87.2
IL-12p70 (pg/ml)	86	24.9	11.6	118.7
IL-1β (pg/ml)	86	4,090	1,476	14,588
Peptidoglycan-stimulated cultures				
IL-10 (pg/ml)	86	468	90	2049
TNF-α (pg/ml)	86	3,391	564	11,334
IL-6 (ng/ml)	86	42.4	11.9	100.6
IL-12p70 (pg/ml)	86	14.3	5.3	64.0
IL-1β (pg/ml)	86	318	29	1,448
Phytohaemagglutinin-stimulated cultures				
IFN-γ (pg/ml)	86	5.2	0.2	55.1
TNF-α (pg/ml)	86	1,846	658	3,472

### Relationship Between Immune Markers and Age, Frailty and Length of Care Home Residence

#### Univariate Analysis

**FIGURE 2:**
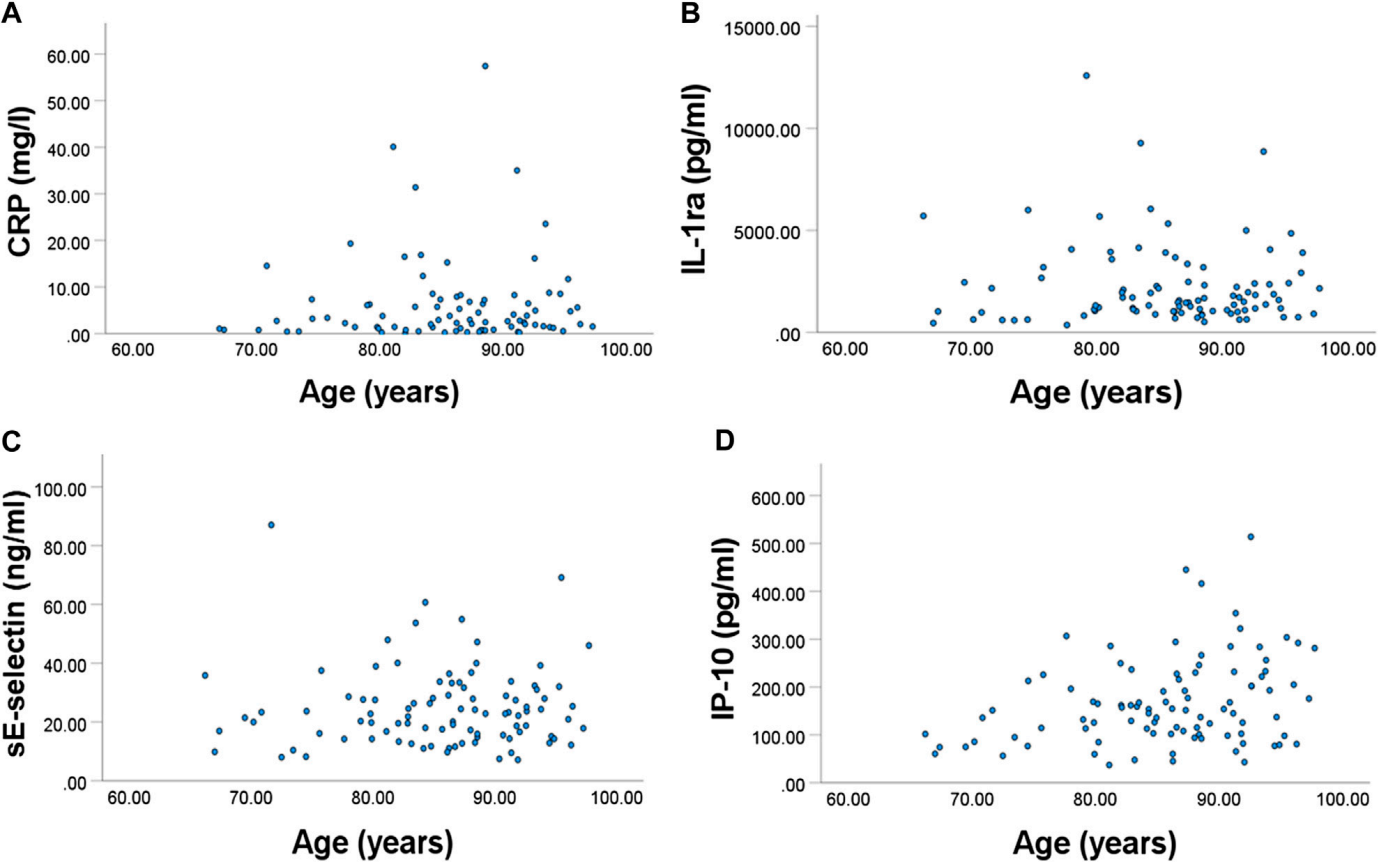
Relationships between frailty index and plasma concentration of **(A)** CRP, **(B)** IL-1ra, (**C)** sE-selectin, and **(D)** IP-10. All were significant.

**FIGURE 3:**
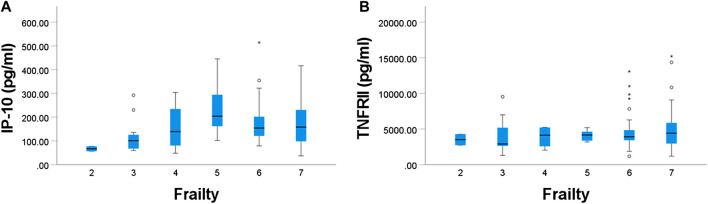
Relationships between age and plasma concentration of **(A)** IP-10 and **(B)** TNF-RII. Both were significant.

Associations of each immune marker with age, frailty and length of care home residence were investigated. In most cases there was no statistically significant association (Supplementary Tables S1–S7). Exceptions were:

- Platelet numbers were positively associated with frailty index (*p* = 0.003).
- Plasma CRP, IL-1ra, sE-selectin, and IP-10 were positively associated with frailty index (*p* = 0.014, 0.023, 0.015, and 0.016, respectively) (Figure 2).
- PGN-stimulated IL-10 production was inversely associated with frailty index (*p* = 0.031).
- Plasma sVCAM-1, IP-10 and TNFRII were positively associated with age (*p* = 0.023, 0.002, and 0.002, respectively) (Figure 3).
- Plasma MCP-1 was positively associated with length of care home residence (*p* = 0.012).

#### Multivariate Analysis

A linear regression model was used to identify the independent contribution of age, frailty and length of care home residence to the various immune parameters as dependent variables (Supplementary Tables S1–S7). Among the parameters included as part of the full blood count, frailty was a significant predictive factor for platelet numbers (adjusted coefficient 0.23 (95% CI: 0.08, 0.37), *p* = 0.002; Supplementary Table S1). Among the immune phenotypes, age was a significant predictive factor for activated monocytes as determined by CD86 expression (adjusted coefficient 2.78 (95% CI: 0.87, 4.70), *p* = 0.005; Supplementary Table S2). Apart from these, none of the covariates was found to contribute significantly to the individual components of the full blood count (Supplementary Table S1) or the immune cell phenotypes (Supplementary Table S2). There were also no predictive associations between the covariates and neutrophil or monocyte phagocytosis (Supplementary Table S3). For immune mediators after stimulation of whole blood cultures, the only predictive association was between frailty and PGN-stimulated IL-10 (adjusted coefficient −0.79 (95% CI: 1.54, −0.04), *p* = 0.038, Supplementary Table S5). Frailty index, age and length of care home residence each independently predicted some plasma immune mediators (Supplementary Table S4). Age was a significant predictor of plasma IP-10 (adjusted coefficient 1.77 (95% CI: 0.61, 2.93), *p* = 0.003), TNFRII (adjusted coefficient 1.76 (95% CI: 0.60, 2.92), *p* = 0.003) and sVCAM-1 (adjusted coefficient 1.19 (95% CI: 0.13, 2.26), *p* = 0.029). Frailty index was an independent predictor of CRP (adjusted coefficient 1.18 (95% CI: 0.34, 2.01), *p* = 0.006), IL-1ra (adjusted coefficient 0.43 (95% CI: 0.00, 0.87), *p* = 0.050), sE-selectin (adjusted coefficient 0.35 (95% CI: 0.05, 0.66), *p* = 0.024) and IP-10 (adjusted coefficient 0.32 (95% CI: 0.32, 0.64), *p* = 0.042). Lastly, length of care home residence was an independent predictor of MCP-1 (adjusted coefficient 0.10 (95% CI: 0.01, 0.19), *p* = 0.026).

## Discussion

Few studies have described immune parameters in older people resident in care homes. Here we describe a selection of immune and inflammatory markers in blood and *ex vivo* immune cell functions in a sample of 184 older people resident in care homes aged 65–102 years and their association with frailty, age and length of care home residence. Almost a third of the participants had low total lymphocyte numbers. Moreover, participants had lower numbers of T lymphocytes and natural killer cells and a lower ratio of CD4^+^ to CD8^+^ T lymphocytes than reported in other studies of older adults (Tavares et al., 2014; Qin et al., 2016). These findings are consistent with the hallmarks of immunosenescence (Boraschi et al., 2013; Pera et al., 2015; Weyand and Goronzy, 2016) and would indicate an increased risk of infections and poor vaccination responses (Crétel et al., 2010; Bektas et al., 2017). Lymphocyte numbers were not associated with age or frailty. This contrasts with the report of Collerton et al. (2012) who found an inverse association of lymphocyte numbers with frailty, assessed using two different models, in 845 85 years olds in the United Kingdom. Furthermore, Bernabeu-Wittel et al. (2019) identified that low lymphocyte numbers were associated with frailty in hospitalized older people with poly-pathologies; they also identified that frailty was a risk factor for mortality at 12 months. In another study, there was an inverse association between frailty score and lymphocyte count in institutionalized older people, but lymphocyte count did not predict hospitalisations or mortality, although frailty did predict mortality (Fernandez-Garrido et al., 2018). Recently, low lymphocyte counts were shown to associate with frailty in patients with cardiovascular disease (Bodolea et al., 2020).

Other associations identified in the current study indicate links between greater frailty and increased inflammation and between increasing age and increased inflammation. The association between frailty and inflammation is consistent with the proposal that frailty is an inflammatory condition (Bruunsgaard and Pedersen, 2003; Angulo et al., 2016), while the associations between age and inflammatory markers or responses are consistent with the concept of inflammageing (Breitbart and Stollar, 2000; Ferrucci and Fabbri, 2018).

A proportion of participants (7.6%) had a platelet count above the upper limit of the reference range. The exact threshold at which platelet numbers become a marker of chronic inflammation has not been clearly defined, but high platelet numbers are related to inflammatory conditions, cancer and infection as well as endothelial dysfunction (Jones, 2016; Haynes et al., 2017) and atherosclerotic plaque formation (Stolla et al., 2016). Moreover, platelet numbers increased across categories of frailty, findings also confirmed through modelling, where frailty emerged as a significant independent predictor of platelet numbers. Recently, Bodolea et al. (2020) found that platelet numbers associate with frailty in patients with cardiovascular disease. Fuentes et al. (2017) report that platelet oxidative stress is a novel marker of cardiovascular risk in frail older people and Starr and Deary (2011) observed increased platelet numbers over a time-frame of 8 years in individuals initially aged over 79 years. The current study did not reveal a significant association of platelet numbers with age. Nevertheless, increased platelet numbers could be a marker of mortality risk through increased frailty. Platelets trigger leukocyte adhesion which favors their aggregation. The mechanism seems to be linked to platelet-induced production of adhesion molecules (Garraud et al., 2012; Jenne et al., 2013).

CD80 and CD86 were used as markers of activated blood monocytes. The linear regression model showed that age was a significant independent predictor of CD86^+^ monocytes over frailty and length of care home residence. Busse et al. demonstrated that monocytes expressing CD86 were increased in elderly individuals (Busse et al., 2015) and concluded this to be a consequence of immunosenescence/inflammageing, as this trait appeared in both a cohort of elderly individuals with dementia and in healthy age-matched controls (Busse et al., 2015).

Phagocytic function has been reported to decline with age leading to a failure to remove foreign antigenic particles and autologous senescent cells (Goronzy and Weyand, 2012; Li, 2013). In the current study, phagocytic function of neutrophils and monocytes was not significantly associated with age, frailty or length of care home residence. These findings do not confirm what has been shown by others where phagocytic function, especially of neutrophils, declined with age (Butcher et al., 2000; Butcher et al., 2001). However, this may be because the current study only investigated older participants. A previous comparison of neutrophil phagocytosis among three age groups (21–36, 38–56, and 62–83 years) found a significant age-dependent reduction in the number of phagocytosed *E. coli* (Wenisch et al., 2000). Thus, that study investigated a much wider age range than in the current study. It is possible that beyond 65 years of age, the alteration in phagocytic activity of neutrophils and monocytes becomes less dramatic than the change between young and older or middle-aged and older individuals.

Previous studies have associated markers of inflammation with different chronic and age-related conditions (e.g., cardiovascular disease and dementia (Assar et al., 2016; Calabrese et al., 2018). Others have reported that age and frailty are factors associated with inflammatory biomarkers (Bruunsgaard and Pedersen, 2003; Angulo et al., 2016; Branas et al., 2018). Indeed, researchers have reported that there is a characteristic “cytokinome” (Costantini et al., 2010) in older people with physical frailty and sarcopenia (Marzetti et al., 2019), suggesting IP-10 to be a marker of frailty and sarcopenia. The current study identified that IP-10 was associated with frailty. In the current study frailty was also an independent predictor of CRP, IL-1ra and sE-selectin. Previous studies have shown that aging is associated with increased concentrations of sICAM-1 and sVCAM-1 (Calder et al., 2017; Calabrese et al., 2018). The current study found that sVCAM-1 concentration had an association with age, as did IP-10 and TNFRII. These findings support the idea that inflammatory pathways are upregulated in aging and in age-related diseases (Cardoso et al., 2018).

Beyer et al. suggest that inflammation is related to muscle wasting, facilitating progression of frailty: in a population of 33 geriatric individuals, those with higher MCP-1 showed a significantly lower grip strength and lower lean body mass (Beyer et al., 2012). Animal research has suggested that MCP-1 is a potential biomarker of biological aging (Yousefzadeh et al., 2018). However, one study reported lower plasma MCP-1 in frail compared with non-frail older care home residents (Seto et al., 2015), while in the current study frailty was not a predictor of MCP-1 concentration.

Other inflammatory markers where frailty appeared as a significant contributory factor over age and length of stay at care home—identified through the regression model—were IL-1ra and the soluble adhesion molecule sE-selectin. IL-1ra opposes the action of pro-inflammatory IL-1 and may be released in an effort to mitigate inflammation. Nevertheless, IL-1ra has been linked as an independent risk factor of morbidity and mortality in the older people resident in care homes (Bruunsgaard and Pedersen, 2003). Upregulation of the expression of adhesion molecules with frailty has been reported (Licastro et al., 2005; Jenny, 2012).

Inflammageing, either low grade or chronic, is commonly linked to morbidity and mortality (Ahmad et al., 2009; Ahmadi-Abhari et al., 2014). Our findings support an association of inflammation with frailty in older people resident in care homes. Inflammageing is a predictor of frailty in elderly (Zhang et al., 2017). Edvardsson et al. have demonstrated that inflammatory markers are related to reduced survival in a follow-up study for one year with frail older people resident in care homes (Edvardsson et al., 2019).

Experiments to assess cellular responses *ex vivo* were performed through whole blood cultures. These experiments allowed assessment of inflammatory and immune mediator production via stimulation of toll-like receptor (TLR)2 and TLR4 with PGN and LPS, respectively, as well as T cell stimulation with PHA. The activation of TLR2 and TLR4 leads to increased production of multiple cytokines (Schwandner et al., 1999; Skinner et al., 2005). Findings herein presented showed that IL-10, TNF-α and IL-1β were potently induced by LPS in comparison to PGN. LPS induced median production values 5-fold higher for IL-10, 3.9-fold higher for TNF-α and almost 12-fold higher for IL-1β when compared with PGN. Furthermore, a superior production of IL-12p70 was induced by LPS when compared with PGN, but the difference was less (two-fold). Lastly, IL-6 was similarly induced by both PGN and LPS. PHA stimulates T cells. The production of TNF-α following PHA stimulation was lower than with LPS and PGN. The potent effects exerted by LPS agree with what has been shown by others (Andersson et al., 1992). The association of health and TLR responsiveness, particularly TLR4, in older people resident in care homes has not been widely explored. McFarlin et al. have suggested that TLR4 appears to have a role in regulating the linkage between cytokine production (IL-1β and TNF-α) and physically active lifestyle regardless of age. In that study, a group of older (60–80 years) and young (18–30 years) adults were categorized as “active” or “inactive.” There was significantly higher production of IL-1β and TNF-α in the inactive group in both young and older people (McFarlin et al., 2006). McFarlin et al. (2006) also reported lower expression of TLR4 in the active group. Similar observations were reported in a group of older women exposed to regular training (McFarlin et al., 2004). Current findings certainly suggest an active TLR4 pathway in the older people resident in care homes according to the cytokine production detected in the cultures following LPS stimulation. A predisposition to active responses of innate immune cells via TLR4, and perhaps other pattern recognition receptors, may be one reason for higher circulating concentrations of inflammatory cytokines in older people, one of the hallmarks of inflammageing.

IL-10 induced by PGN was significantly inversely associated with frailty. IL-10 is an anti-inflammatory cytokine that counterbalances pro-inflammatory responses (Iyer and Cheng, 2012). The older people resident in care homes appeared to show an imbalance in IL-10 and TNF-α.

Our findings may be compared with those of Collerton et al. (2012) who measured a range of immune and inflammatory parameters in 845 85 years olds in the United Kingdom and related these to frailty assessed with two different models. As mentioned earlier, that study reported an inverse association between frailty and lymphocyte numbers which was not observed in the current study. This may represent differences in the characteristics of the participants included in the two studies (all were resident in care homes in the current study whereas this was not the case in Collerton et al. (2012)); age range was 66–102 years in the current study but all participants were aged 85 years in Collerton et al. (2012) or the smaller sample size of the current study. Collerton et al. (2012) also reported positive associations of frailty with total leukocyte and neutrophil counts, which we did not observe. Collerton et al. (2012) reported a positive association between frailty and CRP concentrations, as observed in the current study. They also identified a lack of association of frailty with monocyte, basophil, and eosinophil counts, ratio of CD4^+^ to CD8^+^ lymphocytes, and LPS-stimulated TNF-α and IL-6 production; our observations are consistent with this. Collerton et al. (2012) did not report platelet numbers or plasma concentrations of inflammatory mediators, which were associated with frailty in the current study.

The current study has several strengths. There were few restrictions on participant inclusion. A broad range of immune and inflammatory outcomes were measured, representing several different components of the immune system; these included static measures in blood (full blood count, immune phenotypes, plasma mediators, CRP) as well cell responses after challenge (phagocytosis, blood culture responses to LPS, PGN, and PHA) and components of innate (phagocytosis, blood culture responses to LPS and PGN) and acquired immunity (blood culture responses to PHA). Finally, linear regression modelling was used to identify independent effects of age, frailty, and time of care home residence on the outcomes reported. However, the study also has some limitations. Firstly, not all immune outcomes were available for all 184 participants; this is mainly because some blood samples did not arrive at the laboratory within a predetermined time to assure the viability of the immune assay (Castro-Herrera et al., 2018). Secondly, the samples were from participants in a randomized controlled trial (Owen-Jones et al., 2019; Butler et al., 2020) and this required exclusion of some of the care home residents; thus the findings are not generalisable to all care home residents. Thirdly, we did not collect data on co-morbidities (other than frailty index) or medication use, which might be relevant to immune and inflammatory biomarkers. Finally, since the study was exploratory no power calculation was done, and so non-significant findings must be interpreted with caution, and significant findings interpreted cautiously since we did not correct for the multiple statistical comparisons performed.

## Data Availability

The datasets presented will be linked with clinical data produced as part of the same project. Requests to access the datasets should be directed to David Gillespie GillespieD1@cardiff.ac.uk.
