# Supplementary material for: Relationships Between Age, Frailty, Length of Care Home Residence and Biomarkers of Immunity and Inflammation in Older Care Home Residents in the United Kingdom
Source: Front Aging. 2021 Mar 17;2:599084. doi: 10.3389/fragi.2021.599084 (PMC9261419; doi:10.3389/fragi.2021.599084)
Supplement: Supplementary file 1 [file table1.docx]

Supplementary Material

# Supplementary Tables

## Table S1. Linear regression model for age, length of care home residence and frailty to predict the components of the full blood count in older people resident in care homes

| Variable | | Unadjusted coefficient | 95% Confidence Interval | | p-value | Adjusted coefficient* | 95% Confidence Interval | | p-value |
| --- | --- | --- | --- | --- | --- | --- | --- | --- | --- |
|  |  |  | Lower boundary | Upper boundary |  |  | Lower boundary | Upper boundary |  |
| Neutrophils | Age | 0.11 | -0.58 | 0.8 | 0.756 | 0.11 | -0.61 | 0.83 | 0.764 |
|  | Length in care home | -0.04 | -0.09 | 0.01 | 0.1 | -0.04 | -0.1 | 0.01 | 0.098 |
|  | Frailty | 0.02 | -0.17 | 0.21 | 0.83 | 0.05 | -0.14 | 0.24 | 0.616 |
| Lymphocytes | Age | -0.13 | -0.98 | 0.71 | 0.755 | -0.11 | -1.00 | 0.77 | 0.801 |
|  | Length in care home | -0.02 | -0.09 | 0.04 | 0.445 | -0.02 | -0.09 | 0.04 | 0.530 |
|  | Frailty | -0.07 | -0.31 | 0.16 | 0.535 | -0.06 | -0.30 | 0.18 | 0.636 |
| Monocytes | Age | -0.30 | -0.97 | 0.37 | 0.381 | -0.28 | -0.98 | 0.43 | 0.435 |
|  | Length in care home | -0.03 | -0.08 | 0.02 | 0.196 | -0.03 | -0.08 | 0.02 | 0.263 |
|  | Frailty | -0.07 | -0.26 | 0.11 | 0.449 | -0.05 | -0.24 | 0.14 | 0.628 |
| Total leukocytes | Age | -0.11 | -0.75 | 0.54 | 0.743 | -0.11 | -0.79 | 0.56 | 0.744 |
|  | Length in care home | -0.04 | -0.09 | 0.01 | 0.092 | -0.04 | -0.09 | 0.01 | 0.087 |
|  | Frailty | 0.04 | -0.14 | 0.22 | 0.682 | 0.07 | -0.11 | 0.25 | 0.456 |
| Platelets | Age | 0.06 | -0.32 | 0.73 | 0.442 | 0.16 | -0.38 | 0.69 | 0.567 |
|  | Length in care home | -0.03 | -0.05 | 0.031 | 0.70 | -0.02 | -0.06 | 0.02 | 0.336 |
|  | Frailty | 0.25 | 0.07 | 0.36 | 0.003 | 0.23 | 0.08 | 0.37 | 0.002 |

*Variables are mutually adjusted for each other.

## Table S2. Linear regression model for age, length of care home residence and frailty to predict the components of the immunophenotype in older people resident in care homes

| Variable | | Unadjusted coefficient | 95% Confidence Interval | | p-value | Adjusted coefficient* | 95% Confidence Interval | | p-value |
| --- | --- | --- | --- | --- | --- | --- | --- | --- | --- |
|  |  |  | Lower boundary | Upper boundary |  |  | Lower boundary | Upper boundary |  |
| T cells | Age | -0.14 | -0.62 | 0.34 | 0.566 | -0.14 | -0.63 | 0.35 | 0.574 |
|  | Length in care home | 0.01 | -0.03 | 0.04 | 0.676 | 0.01 | -0.03 | 0.04 | 0.660 |
|  | Frailty | -0.01 | -0.13 | 0.12 | 0.932 | -0.01 | -0.14 | 0.12 | 0.897 |
| Cytotoxic T cells | Age | 0.38 | -0.32 | 1.08 | 0.281 | 0.36 | -0.35 | 1.07 | 0.318 |
|  | Length in care home | -0.02 | -0.07 | 0.03 | 0.445 | -0.03 | -0.08 | 0.03 | 0.315 |
|  | Frailty | 0.11 | -0.08 | 0.30 | 0.239 | 0.12 | -0.07 | 0.31 | 0.201 |
| Activated cytotoxic T cells | Age | -0.23 | -1.17 | 0.70 | 0.620 | -0.28 | -1.23 | 0.66 | 0.556 |
|  | Length in care home | 0.04 | -0.02 | 0.11 | 0.205 | 0.04 | -0.03 | 0.11 | 0.307 |
|  | Frailty | 0.18 | -0.07 | 0.42 | 0.166 | 0.16 | -0.10 | 0.41 | 0.229 |
| Regulatory T cells | Age | 0.83 | -0.77 | 2.42 | 0.307 | 0.83 | -0.79 | 2.45 | 0.311 |
|  | Length in care home | -0.08 | -0.20 | 0.03 | 0.154 | -0.09 | -0.21 | 0.03 | 0.141 |
|  | Frailty | 0.03 | -0.40 | 0.46 | 0.886 | 0.08 | -0.36 | 0.51 | 0.724 |
| Helper T cells | Age | 0.74 | -0.37 | 1.86 | 0.190 | 0.70 | -0.44 | 1.84 | 0.224 |
|  | Length in care home | -0.02 | -0.10 | 0.07 | 0.701 | -0.03 | -0.11 | 0.06 | 0.515 |
|  | Frailty | 0.20 | -0.10 | 0.50 | 0.186 | 0.21 | -0.10 | 0.51 | 0.180 |
| Ratio CD4^+^:CD8^+^ | Age | 0.50 | -0.32 | 1.32 | 0.230 | 0.47 | -0.36 | 1.30 | 0.266 |
|  | Length in care home | 0.00 | -0.06 | 0.06 | 0.973 | -0.01 | -0.07 | 0.06 | 0.853 |
|  | Frailty | 0.12 | -0.10 | 0.34 | 0.266 | 0.12 | -0.10 | 0.34 | 0.293 |
| Monocytes | Age | 0.03 | -0.79 | 0.84 | 0.950 | 0.03 | -0.80 | 0.86 | 0.948 |
|  | Length in care home | -0.02 | -0.08 | 0.04 | 0.509 | -0.02 | -0.08 | 0.04 | 0.502 |
|  | Frailty | 0.00 | -0.21 | 0.22 | 0.977 | 0.02 | -0.21 | 0.24 | 0.881 |
| Activated monocytes (CD80^+^) | Age | 1.33 | -0.39 | 3.06 | 0.129 | 1.31 | -0.44 | 3.06 | 0.142 |
|  | Length in care home | -0.05 | -0.18 | 0.07 | 0.395 | -0.07 | -0.19 | 0.06 | 0.317 |
|  | Frailty | 0.15 | -0.31 | 0.62 | 0.516 | 0.18 | -0.30 | 0.65 | 0.463 |
| Activated monocytes (CD86^+^) | Age | 2.72 | 0.84 | 4.61 | 0.005 | 2.78 | 0.87 | 4.70 | **0.005** |
|  | Length in care home | -0.04 | -0.18 | 0.10 | 0.563 | -0.03 | -0.17 | 0.11 | 0.628 |
|  | Frailty | -0.19 | -0.70 | 0.33 | 0.470 | -0.21 | -0.73 | 0.30 | 0.415 |
| NK cells | Age | -0.02 | -0.80 | 0.76 | 0.966 | -0.02 | -0.81 | 0.77 | 0.958 |
|  | Length in care home | -0.01 | -0.07 | 0.04 | 0.605 | -0.02 | -0.07 | 0.04 | 0.574 |
|  | Frailty | 0.02 | -0.18 | 0.23 | 0.820 | 0.04 | -0.18 | 0.25 | 0.742 |
| B cells | Age | -0.08 | -0.92 | 0.75 | 0.845 | -0.07 | -0.91 | 0.78 | 0.877 |
|  | Length in care home | 0.04 | -0.02 | 0.10 | 0.178 | 0.05 | -0.01 | 0.11 | 0.130 |
|  | Frailty | -0.09 | -0.31 | 0.13 | 0.417 | -0.12 | -0.35 | 0.10 | 0.285 |
| Activated B cells (CD80^+^) | Age | 0.41 | -0.38 | 1.20 | 0.310 | 0.44 | -0.37 | 1.24 | 0.284 |
|  | Length in care home | 0.01 | -0.05 | 0.07 | 0.688 | 0.02 | -0.04 | 0.08 | 0.542 |
|  | Frailty | -0.12 | -0.33 | 0.09 | 0.266 | -0.14 | -0.36 | 0.08 | 0.204 |
| Activated B cells (CD86^+^) | Age | 0.02 | -1.23 | 1.28 | 0.970 | 0.06 | -1.22 | 1.34 | 0.923 |
|  | Length in care home | 0.02 | -0.08 | 0.11 | 0.741 | 0.02 | -0.07 | 0.12 | 0.603 |
|  | Frailty | -0.17 | -0.50 | 0.17 | 0.324 | -0.19 | -0.53 | 0.16 | 0.288 |

*Variables are mutually adjusted for each other.

## Table S3. Linear regression model for age, length of care home residence and frailty to predict phagocytic function in older people resident in care homes.

| Variable | | Unadjusted coefficient | 95% Confidence Interval | | p-value | Adjusted coefficient* | 95% Confidence Interval | | p-value |
| --- | --- | --- | --- | --- | --- | --- | --- | --- | --- |
|  |  |  | Lower boundary | Upper boundary |  |  | Lower boundary | Upper boundary |  |
| Percentage of neutrophils gated with phagocytic activity | Age | -0.10 | -0.50 | 0.31 | 0.641 | -0.08 | -0.49 | 0.33 | 0.693 |
|  | Length in care home | -0.02 | -0.05 | 0.01 | 0.228 | -0.02 | -0.05 | 0.01 | 0.300 |
|  | Frailty | -0.05 | -0.16 | 0.06 | 0.370 | -0.04 | -0.15 | 0.07 | 0.510 |
| Geometric median fluorescence intensity (GMFI) of active neutrophils | Age | 0.16 | -0.43 | 0.75 | 0.596 | 0.14 | -0.46 | 0.75 | 0.637 |
|  | Length in care home | 0.03 | -0.02 | 0.07 | 0.235 | 0.02 | -0.02 | 0.07 | 0.290 |
|  | Frailty | 0.05 | -0.10 | 0.21 | 0.504 | 0.04 | -0.13 | 0.20 | 0.669 |
| Percentage of monocytes gated with phagocytic activity | Age | 0.32 | -0.62 | 1.26 | 0.504 | 0.35 | -0.61 | 1.31 | 0.472 |
|  | Length in care home | 0.00 | -0.07 | 0.06 | 0.899 | 0.00 | -0.07 | 0.07 | 0.959 |
|  | Frailty | -0.12 | -0.38 | 0.13 | 0.328 | -0.13 | -0.39 | 0.13 | 0.314 |
| Geometric median fluorescence intensity (GMFI) of active monocytes | Age | 0.14 | -0.61 | 0.88 | 0.719 | 0.16 | -0.60 | 0.92 | 0.675 |
|  | Length in care home | 0.01 | -0.04 | 0.06 | 0.747 | 0.01 | -0.04 | 0.07 | 0.602 |
|  | Frailty | -0.11 | -0.30 | 0.09 | 0.287 | -0.12 | -0.32 | 0.08 | 0.246 |

*Variables are mutually adjusted for each other.

## Table S4. Linear regression model for age, length of care home residence and frailty to predict plasma CRP and immune mediators in older people resident in care homes

| Variable | | Unadjusted coefficient | 95% Confidence Interval | | p-value | Adjusted coefficient* | 95% Confidence Interval | | p-value |
| --- | --- | --- | --- | --- | --- | --- | --- | --- | --- |
|  |  |  | Lower boundary | Upper boundary |  |  | Lower boundary | Upper boundary |  |
|  | Age | 0.02 | -0.11 | 0.15 | 0.757 | 1.26 | -1.85 | 4.37 | 0.422 |
| CRP | Length in care home | -0.30 | -1.25 | 0.66 | 0.537 | 0.04 | -0.19 | 0.27 | 0.720 |
|  | Frailty | 1.76 | 0.36 | 3.16 | 0.014 | 1.18 | 0.34 | 2.01 | 0.006 |
| sICAM-1 | Age | 0.24 | -0.95 | 1.43 | 0.690 | 0.18 | -1.01 | 1.38 | 0.760 |
|  | Length in care home | 0.03 | -0.05 | 0.12 | 0.431 | 0.02 | -0.06 | 0.11 | 0.589 |
|  | Frailty | 0.22 | -0.10 | 0.53 | 0.172 | 0.20 | -0.12 | 0.52 | 0.226 |
| IL-1ra | Age | 0.40 | -1.24 | 2.05 | 0.627 | 0.28 | -1.34 | 1.89 | 0.735 |
|  | Length in care home | 0.11 | -0.01 | 0.22 | 0.073 | 0.08 | -0.03 | 0.20 | 0.158 |
|  | Frailty | 0.50 | 0.07 | 0.92 | 0.023 | 0.43 | 0.00 | 0.87 | 0.050 |
| sE-Selectin | Age | 0.34 | -0.81 | 1.50 | 0.557 | 0.25 | -0.89 | 1.39 | 0.661 |
|  | Length in care home | 0.03 | -0.05 | 0.12 | 0.410 | 0.02 | -0.07 | 0.10 | 0.697 |
|  | Frailty | 0.37 | 0.07 | 0.67 | 0.015 | 0.35 | 0.05 | 0.66 | 0.024 |
| sVCAM-1 | Age | 1.22 | 0.17 | 2.27 | 0.023 | 1.19 | 0.13 | 2.26 | 0.029 |
|  | Length in care home | 0.03 | -0.05 | 0.11 | 0.487 | 0.02 | -0.06 | 0.10 | 0.595 |
|  | Frailty | 0.13 | -0.16 | 0.41 | 0.374 | 0.09 | -0.19 | 0.38 | 0.516 |
| MCP-1 | Age | -0.32 | -1.55 | 0.92 | 0.613 | -0.40 | -1.60 | 0.80 | 0.510 |
|  | Length in care home | 0.11 | 0.03 | 0.20 | 0.012 | 0.10 | 0.01 | 0.19 | 0.026 |
|  | Frailty | 0.30 | -0.03 | 0.62 | 0.071 | 0.23 | -0.09 | 0.56 | 0.152 |
| IP-10 | Age | 1.87 | 0.68 | 3.06 | 0.002 | 1.77 | 0.61 | 2.93 | 0.003 |
|  | Length in care home | 0.09 | 0.00 | 0.17 | 0.060 | 0.07 | -0.02 | 0.15 | 0.126 |
|  | Frailty | 0.40 | 0.08 | 0.72 | 0.016 | 0.32 | 0.01 | 0.64 | 0.042 |
| TNF-RII | Age | 1.79 | 0.65 | 2.94 | 0.002 | 1.76 | 0.60 | 2.92 | 0.003 |
|  | Length in care home | 0.02 | -0.07 | 0.10 | 0.724 | 0.01 | -0.08 | 0.09 | 0.874 |
|  | Frailty | 0.16 | -0.16 | 0.47 | 0.332 | 0.12 | -0.19 | 0.43 | 0.440 |
| IL-6 | Age | -0.68 | -3.02 | 1.65 | 0.562 | -0.60 | -2.95 | 1.75 | 0.613 |
|  | Length in care home | 0.07 | -0.10 | 0.24 | 0.434 | 0.09 | -0.08 | 0.26 | 0.296 |
|  | Frailty | -0.40 | -1.02 | 0.22 | 0.200 | -0.45 | -1.08 | 0.18 | 0.158 |
| IL-10 | Age | 0.51 | -1.86 | 2.89 | 0.669 | 0.57 | -1.80 | 2.94 | 0.636 |
|  | Length in care home | 0.13 | -0.04 | 0.30 | 0.133 | 0.15 | -0.02 | 0.32 | 0.091 |
|  | Frailty | -0.29 | -0.91 | 0.34 | 0.368 | -0.40 | -1.03 | 0.24 | 0.218 |
| TNF-α | Age | 1.17 | -0.33 | 2.67 | 0.124 | 1.18 | -0.34 | 2.70 | 0.126 |
|  | Length in care home | 0.04 | -0.07 | 0.15 | 0.479 | 0.04 | -0.07 | 0.15 | 0.452 |
|  | Frailty | -0.05 | -0.45 | 0.36 | 0.822 | -0.09 | -0.50 | 0.31 | 0.646 |

*Variables are mutually adjusted for each other.

## Table S5. Linear regression model for age, length of care home residence and frailty to predict immune mediators measured in PGN-stimulated whole blood cultures from older people resident in care homes

| Variable | | Unadjusted coefficient | 95% Confidence Interval | | p-value | Adjusted coefficient* | 95% Confidence Interval | | p-value |
| --- | --- | --- | --- | --- | --- | --- | --- | --- | --- |
|  |  |  | Lower boundary | Upper boundary |  |  | Lower boundary | Upper boundary |  |
| PGN IL-10 | Age | -0.14 | -2.94 | 2.66 | 0.923 | 0.06 | -2.72 | 2.84 | 0.967 |
|  | Length in care home | -0.05 | -0.25 | 0.15 | 0.630 | -0.01 | -0.21 | 0.19 | 0.929 |
|  | Frailty | -0.80 | -1.52 | -0.07 | 0.031 | -0.79 | -1.54 | -0.04 | 0.038 |
| PGN TNF-α | Age | 0.64 | -2.07 | 3.36 | 0.639 | 0.73 | -2.02 | 3.48 | 0.600 |
|  | Length in care home | -0.01 | -0.21 | 0.18 | 0.890 | 0.00 | -0.20 | 0.20 | 0.975 |
|  | Frailty | -0.34 | -1.06 | 0.38 | 0.351 | -0.35 | -1.09 | 0.39 | 0.345 |
| PGN IL-6 | Age | 1.13 | -1.29 | 3.54 | 0.357 | 1.18 | -1.28 | 3.64 | 0.341 |
|  | Length in care home | 0.02 | -0.15 | 0.20 | 0.788 | 0.04 | -0.14 | 0.22 | 0.691 |
|  | Frailty | -0.23 | -0.87 | 0.41 | 0.478 | -0.27 | -0.94 | 0.39 | 0.410 |
| PGN IL-1β | Age | 1.38 | -2.32 | 5.09 | 0.460 | 1.59 | -2.12 | 5.30 | 0.398 |
|  | Length in care home | 0.00 | -0.27 | 0.27 | 0.999 | 0.04 | -0.23 | 0.32 | 0.755 |
|  | Frailty | -0.84 | -1.81 | 0.14 | 0.091 | -0.89 | -1.89 | 0.11 | 0.079 |

*Variables are mutually adjusted for each other.

## Table S6. Linear regression model for age, length of care home residence and frailty to predict immune mediators measured in LPS-stimulated whole blood cultures from older people resident in care homes

| Variable | | Unadjusted coefficient | 95% Confidence Interval | | p-value | Adjusted coefficient* | 95% Confidence Interval | | p-value |
| --- | --- | --- | --- | --- | --- | --- | --- | --- | --- |
|  |  |  | Lower boundary | Upper boundary |  |  | Lower boundary | Upper boundary |  |
| LPS IL-10 | Age | 0.50 | -2.22 | 3.22 | 0.717 | 0.52 | -2.25 | 3.29 | 0.710 |
|  | Length in care home | 0.01 | -0.19 | 0.20 | 0.954 | 0.01 | -0.19 | 0.21 | 0.920 |
|  | Frailty | -0.09 | -0.81 | 0.63 | 0.808 | -0.10 | -0.85 | 0.64 | 0.781 |
| LPS TNF-α | Age | 0.52 | -1.40 | 2.45 | 0.590 | 0.44 | -1.50 | 2.39 | 0.651 |
|  | Length in care home | 0.05 | -0.09 | 0.19 | 0.479 | 0.03 | -0.11 | 0.18 | 0.631 |
|  | Frailty | 0.32 | -0.19 | 0.83 | 0.213 | 0.29 | -0.23 | 0.81 | 0.273 |
| LPS IL-6 | Age | 0.95 | -0.64 | 2.54 | 0.238 | 0.93 | -0.70 | 2.56 | 0.258 |
|  | Length in care home | 0.01 | -0.11 | 0.13 | 0.882 | 0.00 | -0.12 | 0.12 | 0.956 |
|  | Frailty | 0.10 | -0.33 | 0.52 | 0.651 | 0.08 | -0.36 | 0.52 | 0.718 |
| LPS IL-12p70 | Age | 0.20 | -1.81 | 2.22 | 0.842 | 0.25 | -1.80 | 2.30 | 0.809 |
|  | Length in care home | 0.02 | -0.13 | 0.16 | 0.807 | 0.03 | -0.12 | 0.18 | 0.699 |
|  | Frailty | -0.20 | -0.74 | 0.33 | 0.449 | -0.23 | -0.78 | 0.32 | 0.411 |
| LPS IL-1β | Age | 1.74 | -0.59 | 4.08 | 0.141 | 1.69 | -0.69 | 4.07 | 0.161 |
|  | Length in care home | 0.04 | -0.13 | 0.21 | 0.665 | 0.03 | -0.15 | 0.20 | 0.772 |
|  | Frailty | 0.23 | -0.39 | 0.86 | 0.460 | 0.19 | -0.45 | 0.83 | 0.560 |

*Variables are mutually adjusted for each other.

## Table S7. Linear regression model for age, length of care home residence and frailty to predict immune mediators measured in PHA-stimulated whole blood cultures from older people resident in care homes

| Variable | | Unadjusted coefficient | 95% Confidence Interval | | p-value | Adjusted coefficient* | 95% Confidence Interval | | p-value |
| --- | --- | --- | --- | --- | --- | --- | --- | --- | --- |
|  |  |  | Lower boundary | Upper boundary |  |  | Lower boundary | Upper boundary |  |
| PHA IFN-γ | Age | 1.50 | -3.57 | 6.57 | 0.559 | 1.43 | -3.71 | 6.57 | 0.581 |
|  | Length in care home | 0.19 | -0.18 | 0.55 | 0.319 | 0.18 | -0.20 | 0.56 | 0.345 |
|  | Frailty | 0.20 | -1.14 | 1.55 | 0.764 | 0.06 | -1.32 | 1.44 | 0.934 |
| PHA TNF-α | Age | 0.107 | -1.412 | 1.625 | 0.889 | 0.113 | -1.433 | 1.658 | 0.885 |
|  | Length in care home | -0.036 | -0.146 | 0.074 | 0.516 | -0.037 | -0.151 | 0.076 | 0.516 |
|  | Frailty | -0.005 | -0.408 | 0.398 | 0.980 | 0.018 | -0.397 | 0.433 | 0.930 |

*Variables are mutually adjusted for each other.
